# Estrogen receptor alpha activation enhances mitochondrial function and systemic metabolism in high‐fat‐fed ovariectomized mice

**DOI:** 10.14814/phy2.12913

**Published:** 2016-08-31

**Authors:** Dale J. Hamilton, Laurie J. Minze, Tanvi Kumar, Tram N. Cao, Christopher J. Lyon, Paige C. Geiger, Willa A. Hsueh, Anisha A. Gupte

**Affiliations:** ^1^ Center for Metabolic and Bioenergetics Research Houston Methodist Research Institute and Weill Cornell Medical College Houston Texas; ^2^ Houston Methodist Research Institute Houston Texas; ^3^ Houston Methodist Department of Medicine Houston Texas; ^4^ University of Kansas Medical Center Kansas City Kansas; ^5^ Ohio State University Columbus Ohio

**Keywords:** Energy balance, ER*α*, estrogen, mitochondria

## Abstract

Estrogen impacts insulin action and cardiac metabolism, and menopause dramatically increases cardiometabolic risk in women. However, the mechanism(s) of cardiometabolic protection by estrogen remain incompletely understood. Here, we tested the effects of selective activation of E2 receptor alpha (ER *α*) on systemic metabolism, insulin action, and cardiac mitochondrial function in a mouse model of metabolic dysfunction (ovariectomy [OVX], insulin resistance, hyperlipidemia, and advanced age). Middle‐aged (12‐month‐old) female low‐density lipoprotein receptor (*Ldlr*)^*−/−*^ mice were subjected to OVX or sham surgery and fed “western” high‐fat diet (WHFD) for 3 months. Selective ER *α* activation with 4,4′,4″‐(4‐Propyl‐[1H]‐pyrazole‐1,3,5‐triyl) (PPT), prevented weight gain, improved insulin action, and reduced visceral fat accumulation in WHFD‐fed OVX mice. PPT treatment also elevated systemic metabolism, increasing oxygen consumption and core body temperature, induced expression of several metabolic genes such as peroxisome proliferator‐activated receptor gamma, coactivator 1 alpha, and nuclear respiratory factor 1 in heart, liver, skeletal muscle, and adipose tissue, and increased cardiac mitochondrial function. Taken together, selective activation of ER *α* with PPT enhances metabolic effects including insulin resistance, whole body energy metabolism, and mitochondrial function in OVX mice with metabolic syndrome.

## Introduction

Clinical observations have long recognized that premenopausal women are protected from cardiometabolic disorders (Wilson et al. 1985). However, this advantage is lost in women afflicted with diabetes and women who lose ovarian estrogen due to surgical or natural menopause (Ren and Ceylan‐Isik 2004). Loss of estrogen during menopause is associated with central adiposity, insulin resistance, decreased energy expenditure, and greater risk of cardiovascular diseases (CVD) (Wilson et al. 1985; Lovejoy et al. 2008). Animal studies have reported protection of female mice from development of diet‐induced obesity and insulin resistance compared to age‐matched males (Pettersson et al. 2012). Consequently, most studies examining mechanisms of diet‐induced obesity have been performed in male mice. Estradiol (E2), the major biologically active form of estrogen, is known to positively influence insulin action in mice (Ribas et al. 2009). Despite increasing clinical observations of the association of cardiometabolic risk with diabetes and menopause, the mechanism by which E2 regulates insulin action and energy metabolism remains unclear.

E2 regulates energy metabolism by modulating mitochondrial function via genomic and nongenomic effects (Chen et al. 2009), and mitochondrial dysfunction is one of the underlying pathogenic mechanisms for insulin resistance and CVD (Lowell and Shulman 2005). The physiological actions of E2 are primarily mediated by its two receptors, ER*α* and ER*β*, although ER*α* is expressed at greater levels in insulin‐sensitive tissues (Barros et al. 2006b; Ribas et al. 2009). In classical E2‐signaling, two ERs dimerize upon activation by E2, translocate to the nucleus, bind to E2 response elements, and then elicit a transcriptional response. Nonclassical mechanisms independent of DNA binding and involving protein–protein interactions have also been described (Marino et al. 2006). The deficiency of E2 to stimulate ERs, impairment of ER action, or alteration of ER*α*/ER*β* ratios have been implicated in the pathogenesis of the metabolic syndrome (Barros et al. 2006b, 2009; Hevener et al. 2015). ER*α* ^−/−^ mice become obese, glucose intolerant, hyperinsulinemic, and have decreased energy expenditure and locomotion (Bryzgalova et al. 2006; Ribas et al. 2009), which indicates a role for ER*α* in positively regulating metabolism. Recent evidence suggests that ER*α* and ER*β* may have opposing effects on metabolic functions. ER*α* is thought to be the essential ER for most E2‐mediated increases in mitochondrial respiratory chain (MRC) proteins and antioxidant enzymes (O'Lone et al. 2007; Chen et al. 2009). In contrast, ER*β* is believed to down‐regulate certain MRC proteins (Yang et al. 2004; Chen et al. 2009; Lizotte et al. 2009). ER*β* deficiency significantly increases cytochrome c oxidase activity, resistance to superoxide production, and oxidative stress‐associated ATP depletion, implying that ER*β* has a primarily negative regulatory role in mitochondrial function (Yang et al. 2009). The role of ER*α* in mitochondrial function may be especially relevant in the insulin resistant state, particularly in cardiac mitochondria which are under high metabolic stress as a result of increased cardiac dependence on fatty acids and compromised insulin‐stimulated glucose uptake (Stanley et al. 1997). Based on ER*α*'s dominant expression in insulin‐sensitive tissues, including the heart, skeletal muscle, adipose tissue, and liver, and current literature linking its activity to expression of key mitochondrial enzymes (O'Lone et al. 2007; Chen et al. 2009), we chose to examine the effects of an ER*α*‐specific agonist in the deficiency of ovarian E2.

We have previously shown that a high‐fat diet induces alterations in ER expression in skeletal muscle and adipose tissue, which may be associated with decreased glucose metabolism and increased inflammation (Gorres et al. 2011a). We have also shown that ER*α* activation with the potent ER*α* agonist PPT (4,4′,4″‐(4‐Propyl‐[1H]‐pyrazole‐1,3,5‐triyl)), increases insulin‐stimulated glucose uptake and insulin signaling in skeletal muscle (Gorres et al. 2011b). In this study, we investigated the effects of ovariectomy (OVX) and selective ER*α* activation on insulin resistance, metabolism, and mitochondrial function using a novel mouse model that mimics some important aspects of menopause in women: middle‐aged (12‐month‐old) OVX female low‐density lipoprotein receptor‐deficient (*Ldlr* ^*−/−*^) mice fed 3 months of a “western” high‐fat diet (WHFD). Metabolic alterations during menopause are complex because of the multitude of changes that occur during this phase of a woman's life. Both aging and ovarian E2‐deficiency are associated with increased susceptibility to central obesity, dyslipidemia, insulin resistance, and decreased metabolism. OVX alone does not mimic these conditions in mouse models. We therefore chose to use an aging mouse model of E2‐deficiency and obesity with *Ldlr*‐deficiency to mimic the hyperlipidemic metabolic milieu associated with human menopause. In this model, middle‐aged *Ldlr* ^*−/−*^ mice (12 months‐of‐age) are subjected to OVX to induce ovarian E2‐deficiency and fed WHFD that, in conjunction with *Ldlr‐*deficiency and age induces hypercholesterolemia, weight gain, and insulin resistance. We hypothesized that activation of ER*α* with PPT would reduce insulin resistance, increase metabolism, and enhance mitochondrial function in OVX WHFD‐fed middle‐aged *Ldlr* ^*−/−*^ mice.

## Materials and Methods

### Materials

All reagents for mitochondrial studies were purchased from Sigma‐Aldrich, St.Louis, MO. Blood glucose was measured with a one‐touch glucometer. All other materials and the companies they are purchased from are referenced in the methods below.

### Animals and treatments

All mice were housed in microisolation cages under a 12‐h light/dark cycle. Middle‐aged (12‐month‐old) female *Ldlr* ^*−/−*^ mice (Jackson Laboratory) were subjected to bilateral OVX or sham surgery. All mice were fed 12 weeks of a high‐fat, high‐cholesterol “western” diet (WHFD, 45% calories from fat, D12451, Research diets) with negligible phytoestrogen content, which has been previously shown to induce cardiac dysfunction (Wilson et al. 2007). One week after OVX, mice were divided into two groups which received subcutaneously implanted 21‐day‐release pellets (Innovative Research of America, Sarasota, FL) containing vehicle (Veh, PBS) or the ER*α*‐specific agonist PPT (1.88 mg/kg/day). A third group of mice, controls, underwent sham surgeries and were implanted with vehicle pellets. Two days after the pellet implantation, all mice were shifted to WHFD for the duration of the study. Pellets were replaced every 21 days for the duration of the 12‐week study. Mice were weighed weekly and at 11 weeks, WHFD were placed in CLAMS units (Columbus Instruments, Columbus, OH) for metabolic assessment. At 12 weeks WHFD, mice were fasted overnight and killed by cervical dislocation. Hearts and livers were immediately processed to isolate mitochondria for respiratory studies and hearts, livers, white adipose tissue (WAT), and skeletal muscle (gastrocnemius) were frozen for gene expression analyses. All the visceral (gonadal) fat was removed and weighed (grams) for each mouse. Blood glucose was measured at the time of killing using a One Touch Ultra glucose meter (Lifescan, Inc., Milpitas, CA), and the blood was collected for plasma insulin and lipid assessment. Insulin was quantified with an ELISA from EMD Millipore (Billerica, MA), and HOMA‐IR was calculated from the insulin and glucose values. Plasma lipids were measured by the Mouse Phenotyping core of the University of Cincinnati. All animal protocols were approved by the Houston Methodist Research Institute's Institutional Animal Care and Use Committee and were compliant with the UK regulations on animal experimentation.

### Mitochondrial function analyses

Mitochondrial respiratory function was assessed with Oroboros high‐resolution respirometry (Innsbruck, Austria) using isolated cardiac mitochondria (0.2 mg/mL). Mitochondria were isolated from freshly excised hearts as described before using a differential centrifugation procedure (Gupte et al. 2013, 2014). Briefly, hearts were washed with an ice‐cold isolation buffer (Buffer A: 220 mmol/L mannitol, 70 mmol/L sucrose, 5 mmol/L MOPS, pH 7.4), and tissue was cut into small pieces, homogenized in buffer A, and centrifuged (800 rpm for 10 min at 4°C). The supernatant was centrifuged at 15,871 *g* for 10 min and the resulting pellet was resuspended in cold buffer B (2 mmol/L EGTA, 0.2% free fatty acid‐free‐BSA in buffer A) and again centrifuged at 13,000 rpm for 10 min. The pellet was rinsed with buffer A, centrifuged for the same time and speed, after which the pellet was resuspended in 30 *μ*l of cold buffer E (0.05 mmol/L of EGTA in buffer A).

A multiple substrate protocol using saturating levels of ADP (for glutamate‐malate, succinate) for maximal OXPHOS (oxidative phosphorylation) rates and a single substrate protocol with unsaturating levels of ADP (for palmitoyl carnitine, pyruvate‐malate) were used, modified from previously described protocols (Pesta and Gnaiger 2012; Gupte et al. 2013). Mitochondria were suspended in MiRO5 medium (0.5 mmol/L EGTA, 3 mmol/L MgCl_2_.6H_2_O, 60 mmol/L K‐lactobionate, 2 mmol/L taurine, 10 mmol/L KH_2_PO_4_, 20 mmol/L HEPES, 110 mmol/L Sucrose, 1 g/L fatty acid free bovine serum albumin, pH 7.1). Substrates glutamate‐malate (GM, 5 mmol/L), ADP (4 mmol/L for saturation), and succinate (5 mmol/L) were sequentially added to measure maximum oxidative phosphorylation capacity. ATP‐synthase‐dependent coupled respiration was determined by quantifying the decline in oxygen consumption after addition of oligomycin (1.5 *μ*g). For the single substrate protocols, either pyruvate‐malate (PM, 5 mmol/L each) or palmitoyl carnitine‐malate (PC, 5 mmol/L each) were added, followed by 100 *μ*mol/L ADP, and followed till exhaustion. State 2 respiration rate (ADP‐independent, substrate‐dependent) was determined as respiration with substrate alone, state 3 as ADP‐supported respiration, and state 4_o_ as ATP‐synthase‐independent respiration after oligomycin addition. Respiratory control ratios (RCR), an index of respiratory efficiency, were calculated as the ratio of state 3 (ADP‐supported) to state 4_o_ (oligomycin‐inhibited) or state 4 achieved by ADP exhaustion for single substrates. All readings were normalized for mitochondrial protein content as determined by Lowry assay.

### Metabolic assessment

Cohort 2 mice were singly housed for five consecutive days in study week 10 and starting and ending diet was weighed each day to calculate average food intake. Core temperature was measured at week 10 using sterilized rectal temperature probes. Petroleum jelly was used as a lubricant, and the probe was inserted for 1 min and repeated five consecutive days for an average reading. At week 11, cohort 2 mice were singly housed and acclimatized in CLAMS chambers for 3 days, then assessed for 2 days (two light and two dark cycles) to determine baseline metabolic rates. The CLAMS chambers house mice individually and are equipped with controlled airflow and sensors for monitoring oxygen and carbon dioxide entering and exiting the cage. VO_2_ consumption and VCO_2_ release was graphed as volume of gas per kg body weight (mL/kg) at standard temperature and pressure versus time for two light–dark cycles. Respiratory exchange ratio (RER) was calculated as ratio of VCO_2_/VO_2_ and energy expenditure was calculated using the abbreviated Weir equation as previously described (Smemo et al. 2014).

### RNA purification, cDNA synthesis, and qRT‐PCR

RNA from heart, liver, WAT, and skeletal muscle was isolated using RNeasy kits (Qiagen, Hilden, Germany), reversed‐transcribed into cDNA with High Capacity cDNA Reverse Transcription kits (Applied Biosystems, Grand Island, NY), and analyzed for candidate gene expression using Taqman PCR Core reagent kits and gene‐specific primer/probe sets (Applied Biosystems). All data are shown as delta Ct values. Candidate gene expression was normalized to *Ppia* (cyclophilin) expression (Collins et al. 2009). *Ppia* was chosen as the reference gene since *Ppia* expression did not vary with diet or treatment.

### Statistics

Mann–Whitney nonparametric test was performed to identify differences in the following pair‐wise comparisons: Sham versus OVX + Veh, OVX + Veh versus OVX + PPT, Sham versus OVX + PPT. A two‐tailed *α *= 0.05 was used as the significance cutoff for all tests. Data are presented as means ± SEM and sample sizes are reported in figure legends. GraphPad Prism 5.0 software (GraphPad Software, Inc., La Jolla, CA) was used for all statistical analyses.

## Results

### ERα activation with PPT prevents weight gain, reduces insulin resistance, and improves metabolic function in WHFD‐fed aged OVX mice

**Figure 1:**
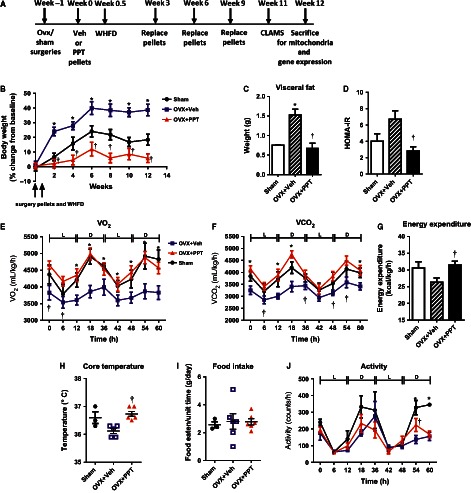
PPT treatment improves systemic metabolism, prevents weight gain, and improves insulin sensitivity on a “western” high‐fat diet (WHFD). (A) Shows the experimental design. (B) Weekly body weight change, (C) visceral fat weight, (D) HOMA‐IR values, (E) Whole body oxygen consumption, (F) CO_2_ release during and light (L) and dark (D) cycle, (G) Energy expenditure, (H) Core body temperature, (I) food intake, and (J) ambulatory activity of sham, OVX + Veh and OVX + PPT mice after 11 weeks of WHFD. (Data shown as mean ± SEM; *N* = 3–4 sham, 4–5 OVX + Veh, and 5–6 OVX + PPT. *P < 0.05 vs. sham, †*P* < 0.05 OVX + PPT vs. OVX + Veh by Mann–Whitney test).

To test whether specific activation of ER*α* affects metabolism and insulin action, middle‐aged OVX mice were implanted with subcutaneous osmotic pellets administering vehicle or the ER*α*‐specific agonist PPT and fed WHFD for 12 weeks (Fig. 1A). Although mice from the three treatment groups had similar body weights at the start of the study, vehicle‐treated OVX (OVX + Veh) mice gained significantly more weight than vehicle‐treated sham‐surgery control (sham) mice, while PPT‐treated OVX (OVX + PPT) mice were strongly resistant to weight gain with WHFD throughout the study (Fig. 1B). Correspondingly, OVX + PPT mice had significantly less visceral fat than OVX + Veh mice, similar to the sham mice (Fig. 1C). The HOMA‐IR index, a measurement of insulin resistance, was significantly lower in OVX + PPT mice than OVX + Veh mice, suggestive of improved insulin sensitivity with PPT (Fig. 1D). Taken together, PPT treatment prevents weight gain and the onset of insulin resistance with a WHFD in OVX mice.

To investigate the effect of PPT to prevent WHFD‐induced weight gain, the mice were examined for differences in metabolism, food intake, and activity after 11 weeks of WHFD. OVX + Veh mice exhibited decreased metabolic activity versus sham mice, with reduced O_2_ consumption and CO_2_ release rates (Fig. 1E–F), both of which were completely normalized by PPT treatment. The area under the curve (AUC) for VO_2_ and VCO_2_ were as follows: sham 269,005.5 ± 13,210.5, 226,168 ± 16,507; OVX + Veh 226,905.5 ± 11,042.7, 195,513.5 ± 9179.5; OVX + PPT 273,450.4 ± 9245.8, 242,170.4 ± 10,013.5, with *P* < 0.05 for OVX + Veh versus OVX + PPT. This data suggested that OVX was responsible for decreased metabolic activity in the OVX + Veh mice and that ER*α* restores metabolic activity in these mice. No significant differences were detected in the RERs (ratio of VCO_2_ to VO_2_) of these groups. RERs of sham mice (0.85 ± 0.01) and OVX + Veh mice (0.86 ± 0.01) were indicative of mixed carbohydrate and lipid oxidation, although a trend toward a higher RER for OVX + PPT mice (0.89 ± 0.01) was suggestive of a slight increase in carbohydrate metabolism. Total energy expenditure (EE) tended to decrease in OVX mice but significantly increased by PPT treatment (Fig. 1G). Consistent with these results, OVX + Veh mice tended to exhibit lower body temperatures than sham and significantly less than OVX + PPT mice (Fig. 1H). However, increased O_2_ consumption and CO_2_ release rates in OVX + PPT mice occurred independent of increased food intake (Fig. 1I). There was no overall difference in ambulatory activity among the three groups: AUC Sham 13,816 ± 2909; OVX + Veh 9175.25 ± 1363.4; OVX + PPT 9659.6 ± 1162.9 (Fig. 1J). Taken together, these results suggested that PPT increased energy expenditure and body temperature in OVX + Veh mice, which may partially explain the differential weight gain of OVX + Veh and OVX + PPT mice.

### Expression of mitochondrial and metabolic genes is enhanced by PPT treatment

We examined the expression of key genes for E2 signaling, mitochondrial biogenesis, inflammation, and glucose and lipid metabolism and in skeletal muscle, liver, WAT, and heart. We also examined metabolic genes important to specific tissues, such as Pepck in the liver.

**Figure 2:**
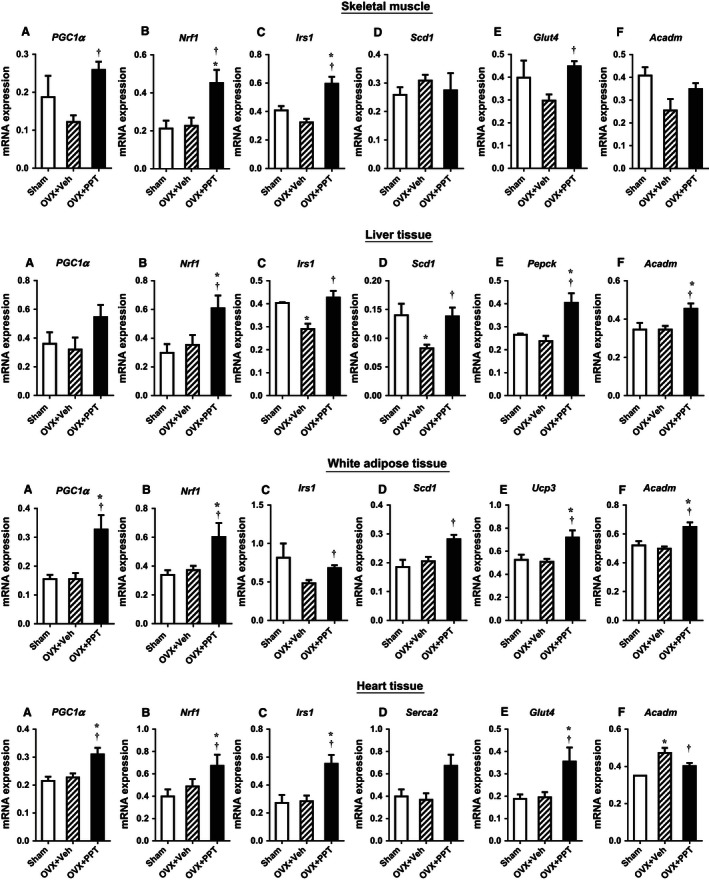
PPT treatment impact mitochondrial and glucose and fatty acid metabolism genes in OVX mice. mRNA expression for heart, liver, skeletal muscle, and white adipose tissue as measured by quantitative RT‐PCR. (Data shown as mean ± SEM; *N* = 3 sham, 4 OVX + Veh, and 6 OVX + PPT. †*P* < 0.05 vs. OVX + Veh by Mann–Whitney test).

In the gastrocnemius skeletal muscle (Fig. 2 skeletal muscle A–F), expression of mitochondrial regulators peroxisome proliferator‐activated receptor gamma, coactivator 1 alpha (*Pgc1α*), and nuclear respiratory factor 1 (*Nrf1*) were induced with PPT. Higher levels of *Pgc1a* can enhance mitochondrial biogenesis as well as oxidative processes, such as fatty acid oxidation. Expression of the glucose metabolism genes *Irs1* and *Glut4* were induced by PPT, but fatty acid metabolism genes steroyl CoA desaturase 1 (*Scd1*) and acyl CoA dehydrogenase, medium chain (*Acadm*) remained unchanged.

In the liver (Fig. 2, Liver tissue A–F), *Pgc1α* did not change significantly. PPT increased the expression of *Nrf1*, whereas OVX + Veh liver had decreased *Irs1* and *Scd1* expression which was rescued by PPT treatment. PPT also induced expression of phosphoenolpyruvate carboxykinase (*Pepck*) and *Acadm* in the liver.

In WAT (Fig. 2, WAT A–F), *Pgc1α* and *Nrf1* were induced by PPT treatment. *Irs1* and *Scd1* were induced with PPT treatment compared with OVX + Veh mice. Expression of uncoupling protein 3 (*Ucp3*) and *Acadm* were increased in OVX + PPT mice compared to sham and OVX + Veh mice.

In the heart tissue (Fig. 2, Heart tissue A–F), we found that PPT treatment increased mRNA expression levels of *Pgc1a* and *Nrf1*. PPT induced expression of insulin receptor substrate 1 (*Irs1*), tended to increase expression of sarco/endoplasmic reticulum Ca^2+^ ATPase (*Serca2*), and increased expression of glucose transporter *Glut4*. Expression of the fatty acid oxidation gene *Acadm* was increased in OVX + Veh mice and decreased to sham levels with PPT.

These data suggest that PPT may increase metabolism and insulin sensitivity by inducing tissue‐specific changes in the expression of mitochondrial, insulin signaling, glucose transport, and fatty acid metabolism genes.

### PPT increases expression of mitochondrial genes and enhances mitochondrial respiratory function in the heart

**Figure 3:**
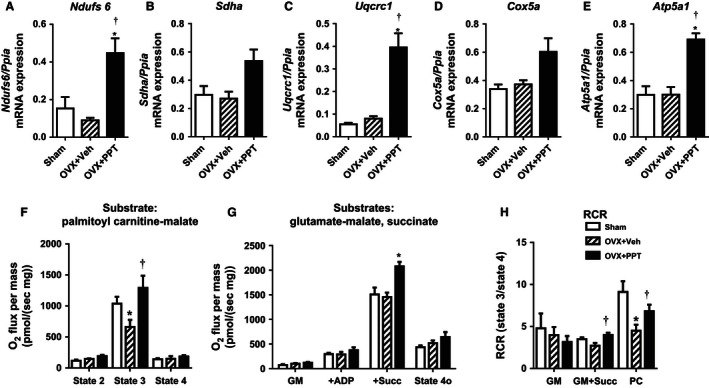
PPT treatment enhances cardiac mitochondrial function. Cardiac mRNA expression of the (A–E) mitochondrial genes N*dufs6*,* Sdha*,* Uqcrc1*,* Cox5a*,* Atp5a1* were measured by quantitative RT‐PCR. (Data shown as mean ± SEM; *N* = 3 sham, *N* = 4 OVX + Veh, and *N* = 6 OVX + PPT. *P < 0.05 vs. sham, †*P* < 0.05 vs. OVX + Veh by Mann–Whitney test). Oxygen consumption rates of cardiac mitochondrial isolated from sham, OVX + Veh, and OVX + PPT‐treated mice. (F) Respiration rates for palmitoyl carnitine‐malate (PC) were measured at state 2, state 3, and state 4; (G) Respiration of cardiac with glutamate‐malate alone (GM; state 2 ADP‐independent), state 3 ADP‐supported respiration with GM + ADP (+ADP) and succinate (+Succ), and state 4_o_ ATPase‐independent respiration after oligomycin addition; (H) Respiratory control ratios (RCR) were calculated for GM, GM + Succ, and PC. (Data shown as mean ± SEM; *N* = 4 for sham, OVX + Veh, and OVX + PPT. *P < 0.05 vs. sham, †*P* < 0.05 vs. OVX + Veh by Mann–Whitney test).

Given the high energy demand of the cardiac muscle, efficient mitochondrial function is crucial for normal cardiac function. However, little is known about the effects of PPT on cardiac bioenergetics. We therefore chose to examine the effects of PPT on cardiac mitochondrial gene expression and respiratory function as a representation of its mitochondrial effects. PPT treatment significantly induced cardiac expression of *Ndufs6* (complex I), *Uqcrc1* (complex III), and *ATP5a1* (complex V) and tended to increase *Sdha* (complex II) and *Cox5a* (complex IV) (Fig. 3A–E) in the heart.

To assess the effects of OVX and PPT on mitochondrial function, mitochondria were isolated from left ventricular (LV) wall tissue of sham, OVX + Veh, and OVX + PPT mice. Cardiac mitochondria from each group demonstrated similar respiration when incubated with palmitoyl‐carnitine and malate in the absence of ADP (state 2) and upon ADP exhaustion (state 4, Fig. 3F). However, OVX + Veh mice revealed a reduction in ADP‐dependent respiration with nonsaturation ADP concentrations that was fully reversed in the mitochondria of OVX + PPT mice (Fig. 3F). In a multisubstrate protocol, cardiac mitochondria from OVX + PPT mice also significantly differed from sham and OVX + Veh mice by demonstrating greater maximal OXPHOS respiration in response to succinate + GM + saturating levels of ADP (+Succ), but not GM + ADP (+ADP). There were no differences in state 2 ADP‐independent (GM) or state 4o ATPase‐independent respiration (state 4_o_) (Fig. 3G). Respiratory efficiency (RCR = ADP‐coupled state 3/ADP‐limited state 4_o_ respiration), a measure of high efficiency conversion of O_2_ consumption to ATP, of OVX + Veh mitochondria was decreased with palmitoyl‐carnitine + ADP (PC) and tended to decrease with glutamate malate + succinate + ADP (GM + Succ), but was fully reversed in OVX + PPT mitochondria (Fig. 3H). Thus, increased metabolism in the PPT‐treated mice is associated with increased cardiac mitochondrial function. Reduced fatty acid oxidative capacity in cardiac mitochondria of OVX mice may have implications for cardiac function, since fatty acids are the primary energy source of the heart. However, we did not see changes in cardiac contractile function with echocardiography with OVX or PPT given the short duration of the protocol (data not shown). However, the LV volume was reduced in OVX mice with a tendency to increase with PPT (Sham 79.37 ± 11.10; OVX + Veh 52.30 ± 12.64; PPT 69.01 ± 4.26; *P* < 0.05 Sham vs. OVX + Veh, *P* = 0.07 OVX + Veh vs. OVX + PPT), indicating the possibility of diastolic dysfunction with OVX that tended toward improvement with PPT.

## Discussion

Despite the long‐standing recognition of the protection against insulin resistance and heart disease in premenopausal women in contrast to men of the same age, the role of E2 in cardiac energy metabolism remains poorly understood. The aim of this study was to determine the impact of selective ER*α* activation on metabolism, insulin resistance, and mitochondrial function in an OVX mouse model that mimics important aspects of metabolic syndrome and aging that are characteristic to menopause. Our data show that selective ER*α* activation prevented WHFD‐induced weight gain and insulin resistance, improved systemic metabolism, and enhanced mitochondrial function in the heart from OVX mice. PPT‐induced expression of *Pgc1a* and *Nrf1* may be one of the potential mechanisms underlying improved mitochondrial function and metabolism. These results suggest that ER*α* activation is important in metabolism, energy balance, insulin sensitivity, and mitochondrial function.

Previous studies in adipose tissue and skeletal muscle have shown that ER*α* positively regulates insulin action (Barros et al. 2006a,b; Ribas et al. 2009; Gorres et al. 2011a,b), whereas ER*β* may have diabetogenic effects (Barros et al. 2009). The underlying mechanisms of ER*α*‐mediated protection of insulin sensitivity include regulation of inflammation (Ribas et al. 2009; Gorres et al. 2011a), Glut4 expression (Barros et al. 2006b; Gorres et al. 2011b), insulin signaling and insulin‐mediated glucose uptake (Gorres et al. 2011b), and oxidative metabolism (Ribas et al. 2009). We find that selective ER*α* activation with PPT prevents WHFD‐induced insulin resistance in OVX mice, and also completely blocks weight gain associated with development of insulin resistance, possibly by preventing a WHFD‐induced decrease in systemic metabolism. Tissue‐specific gene expression such as the increase in *Irs1* and *Glut4* expression in the heart and skeletal muscle with PPT treatment suggests that ER*α* may have direct effects on insulin signaling. PPT also enhanced the expression of the fatty acid oxidation gene *Acadm* in the liver and WAT, suggesting improved flux and oxidation of fatty acids. Therefore, it is likely that insulin action is improved by a combination of the effects of PPT on increasing metabolism, mitochondrial substrate oxidation, reducing adiposity, and direct effects on insulin signaling in heart, liver, muscle, and adipose tissue.

Cardiac mitochondrial function was enhanced by PPT in OVX mice particularly for PC, which may result from a direct effect of ER*α* activation to induce *Pgc1a*,* Nrf1*, and components of the MRC (Fig. 3), although other mechanisms independent of *Pgc1a*, such as mitochondrial antioxidant system, may be altered by ER*α* treatment to impact cardiac mitochondrial activity. It is also likely that some of the effects of PPT on cardiac mitochondrial function may be secondary to reduced adiposity. The PPT effect on cardiac *Pgc1a* and *Nrf1* expression was not obviously regulated by body weight, since it was unaltered by the additional weight gain in OVX + Veh versus sham mice, while it increased with reduced weight gain in OVX + PPT mice. Furthermore, PPT‐induced ER*α* activation produced a robust increase in O_2_ consumption and CO_2_ release in OVX, without altering substrate preference as seen by no change in RER. This suggests overall reversal of metabolism from OVX rather than substrate switching. Similar to previous studies with younger OVX mice (Laudenslager et al. 1980), we found that OVX increased fat mass, body weight, and insulin resistance. However, unlike previous studies, OVX did not increase food intake in our mice, since hyperphagia with OVX is characteristic of rats but not mice (Witte et al. 2010). We and others have shown improvement of insulin action in models of insulin resistance treated with PPT (Lundholm et al. 2008; Gorres et al. 2011a,b), and hormone replacement therapy has been shown to improve insulin resistance in humans (Crespo et al. 2002).

E2 appears to have little effect on mitochondrial ATP production under basal conditions, but may have more robust effects under stress conditions (Wang et al. 2001). Indeed, in our model, aging and WHFD‐induced insulin resistance may provide the additional stress stimuli that exacerbate the effects of OVX on mitochondria. Age has been reported to enhance OVX effects in another report (Hunter et al. 2007), which found that elderly OVX rats exhibited more severely impaired functional recovery and greater infarct size following ischemia reperfusion than those observed with aging or OVX alone. Similar synergistic detrimental effects of aging and ovarian E2‐deficiency are observed in postmenopausal women (Lancaster et al. 2012). We therefore chose to use “middle‐aged” female mice and performed OVX. Although OVX may also remove other hormones secreted by the ovaries, the loss of E2 is the primary hormone with metabolic implications that is affected. Furthermore, while contractile dysfunction (normal ejection fraction) was not found in these mice, we did not assess diastolic function. However, it is likely that the OVX mice have diastolic dysfunction with preserved ejection fraction (reduced LV volume), commonly seen in elderly postmenopausal women (Morgan 2013; Dahiya et al. 2015; Fontes‐Carvalho et al. 2015).

This study is not without its limitations. Given the laborious and time‐sensitive nature of mitochondrial function assessment on isolates, we were not able to concurrently assess mitochondrial function in skeletal muscle along with the heart. Future studies should compare the effects of PPT on muscle mitochondrial function to determine if that underlies the improvement in insulin resistance. Another limitation of the study is that reduction in mitochondrial function or gene expression with OVX was not as dramatic as the increase in mitochondrial function or gene expression with PPT. This is likely due to the absence of negative regulation by ER*β* that may be coactivated with E2. Also, all the mice in the study are middle‐aged, HFD‐fed, and *Ldlr* ^*−/−*^, thus even the sham mice are insulin resistant and hyperlipidemic, and have some degree of mitochondrial dysfunction and depression of metabolic genes. Therefore, the differences between sham and OVX may not be as profound, and the effects of PPT to improve the insulin resistance, metabolism, and mitochondrial function are better than those of the sham mice. A longer period of OVX‐induced ovarian E2‐deficiency may potentially develop further down‐regulation of MRC genes. Also, PPT may not simply restore the effects of E2 in OVX mice, but does more; and the mechanisms of OVX‐induced decrease in mitochondrial function and PPT‐induced restoration may not be the same. Importantly, the 12 weeks of PPT treatment did not show any toxicity but only improvements in weight, metabolism, and insulin sensitivity.

In conclusion, our results suggest a beneficial role for ER*α* activation on whole body and cardiac energy metabolism, even in the presence of underlying metabolic syndrome. Our data suggest that PPT‐mediated ER*α* activation enhances systemic oxidative metabolism, increases mitochondrial function, and improves insulin resistance in a mouse model of dysregulated metabolism due to aging, WHFD, hyperlipidemia, and ovarian E2 deficiency. Future studies are required to identify agonists that selectively activate ER*α* only in specific target tissues. Future studies should also determine the molecular pathway(s) by which ER*α* induces *Pgc1α* and *Nrf1*, and determine whether these mitochondrial changes with ER*α* activation in the heart are protective against heart failure.

## Conflict of Interest

The authors have no conflicts of interest to disclose.
